# Does internet use promote the health of empty-nest older adults in rural China? The mediating role of social participation using a propensity score matching approach

**DOI:** 10.3389/fpubh.2024.1436525

**Published:** 2024-11-22

**Authors:** Li Shen, Yawen Zheng, Mengting Wang, Hong Pan, Wenqian Jian, Xudong Yang, Wei Wang, Li Chen

**Affiliations:** ^1^Department of Psychiatry, The Third People's Hospital of Huzhou Municipal, The Affiliated Hospital of Huzhou University, Huzhou, Zhejiang, China; ^2^Lishui Second Hospital, Wenzhou Medical University, Lishui, Zhejiang, China; ^3^School of Mental Health, Wenzhou Medical University, Wenzhou, Zhejiang, China; ^4^Cixi Biomedical Research Institute, Wenzhou Medical University, Ningbo, China; ^5^The Affiliated Wenzhou Kangning Hospital, Wenzhou Medical University, Wenzhou, Zhejiang, China

**Keywords:** older adults, internet use, physical health, cognitive health, depression, social participation, propensity score matching

## Abstract

**Background:**

The rapid growth of internet use among older adults in rural China offers a unique opportunity to examine its potential impact on their health. This study seeks to explore the relationship between internet use and the emotional, physical, and cognitive health of empty-nest older adults in rural China, while also considering the mediating role of social participation in this relationship.

**Methods:**

A total of 3,478 empty-nest older adults were selected from the China Health and Retirement Longitudinal Survey (CHARLS) in 2020. Among them, 820 individuals were included in the study using a propensity score matching (PSM) method to reduce the impact of selection bias. The participants' depression, physical health, and cognitive health were measured using the Center for Epidemiological Studies Depression Scale (CES-D), the Activities of Daily Living (ADL) scale, and a composite cognition score, respectively. Social participation was assessed based on engagement in various activities over the past month.

**Results:**

After controlling for confounding factors through PSM, it was found that older adults who used the internet exhibited significantly lower level of depression and higher physical health compared to older adults who did not use the internet. However, there was no significant difference in cognitive health between the two groups. The mediating effect analysis revealed that social participation partially mediated the relationship between internet use and physical health, but not emotional health.

**Conclusion:**

The study suggests that internet use can improve emotional and physical health of older adults living in rural China without children at home, with social participation being a mediator in the relationship between internet use and physical health. Future research should explore the underlying mechanisms and develop targeted interventions to promote internet use and social engagement in this population.

## 1 Introduction

The global aging issue is receiving increasing attention, with China seeing the highest growth rate of aging population globally (1). According to the National Bureau of Statistics of China, the number of individuals aged 60 and above in China had reached 280 million by the end of 2022, accounting for 19.8% of the total population. A large portion of this demographic consists of empty-nest older adults residing in rural areas, totaling ~67 million individuals, or one-fourth of the older population (2).

In China's older population, empty-nest rural older adults represent a group that warrants particular attention. Defined as individuals aged 60 and above living in rural areas without children or family members (3), these older adults differ significantly from their urban counterparts. They encounter unique challenges, including limited access to healthcare services, social support, and technological infrastructure. encounter unique challenges such as social isolation, loneliness, and limited healthcare access (4–7). These disparities can profoundly impact their emotional, physical, and cognitive health (8–10). Furthermore, China is undergoing rapid aging and urbanization, resulting in a substantial migration of younger generations from rural to urban areas. This migration leaves many older adults in rural regions without the support of their children, thereby exacerbating the challenges they face (11).

The absence of close relatives may result in feelings of loneliness, isolation, and despair, heightening the risk of depression (12, 13). For example, a study conducted in rural China found that the prevalence of depressive symptoms among empty-nest older adults was 44.2%, which was significantly higher than that of non-empty-nest older adults (26.3%) (14, 15). Additionally, the lack of mental stimulation and social interaction can lead to cognitive decline, affecting memory, attention, and executive function, potentially increasing the risk of dementia (16, 17). A meta-analysis of studies conducted in China found that empty-nest older adults (22.9%, 95% CI: 18.9–27.3%) had a higher risk of cognitive impairment compared to non-empty-nest older adults (19.3%, 95% CI: 15.9–23.0%) (18). Moreover, the absence of assistance in daily activities may contribute to a decline in physical function, impacting mobility, strength, and balance, and raising the risk of physical inactivity and chronic diseases such as cardiovascular disease, diabetes, and cancer (19–21). Therefore, it is crucial to investigate the factors contributing to these health challenges and develop effective interventions to improve the emotional, cognitive, and physical health of empty-nest older adults in rural China.

The rapid development of the internet has been recognized as a potential strategy to address challenges and promote the health of empty-nest rural older adults (22). In China, digital technology, notably smartphones, has seen increased adoption among older age groups (23). As of 2021, more than half of older adults aged 65–69 use smartphones, with 31.2% of those aged 70–79 and 1.3% of those aged 100 and above also using smartphones (24). Additionally, according to recent statistics, the overall internet penetration rate among older adults in China has been steadily increasing. For instance, the China Internet Network Information Center (CNNIC) reported that as of 2021, the number of older adult Internet users aged 60 years or older in China reached 119 million, and the Internet penetration rate reached 43.2%, indicating a significant rise in internet use among the older adult population (25). However, the digital divide remains more pronounced in rural areas (26). Exploring how internet use can benefit rural empty-nest older adults is essential for bridging this divide and promoting equitable access to technology. China's rural revitalization strategy aims to narrow the urban-rural gap, and understanding the impact of internet use on the health of rural empty-nest older adults can inform policy decisions and interventions. The Social Support Theory posits that social relationships and support can buffer against stress, promote health and wellbeing, and encourage healthier behaviors (27). In the context of internet use, online communication and engagement can expand social networks and provide additional sources of social support, which can be particularly beneficial for empty-nest older adults in rural areas who may otherwise feel socially isolated. Previous studies have indicated that internet use can have a positive impact on the health of older adults (23, 28, 29). For instance, a study in America revealed that internet use was linked to improved self-rated health and reduced depressive symptoms in older adults (30). Similarly, a study in China demonstrated that internet use was associated with enhanced cognitive function and decreased feelings of loneliness among older adults (31). Nonetheless, there have been conflicting findings in the literature. Some studies have found no significant relationship between internet use and health outcomes, while others have even reported negative associations (32, 33). For instance, a study in the United States showed that internet use did not correlate with improved physical or mental health, and excessive internet use was linked to adverse health consequences like depression and sleep issues (34).

Inconsistent findings on the relationship between internet use and health in older adults may be attributed to the lack of control for confounding variables. These extraneous factors can influence both internet use and health outcomes, leading to a potentially misleading association. Variables such as age, gender, education level, marital status, number of children, chronic disease, smoking, alcohol consumption, physical activity, and children's support can impact both internet use and health outcomes in older adults (32, 35–37). For instance, younger older adults are more inclined to use the internet compared to their older counterparts, and health issues related to age can affect internet use (38). Higher levels of education are associated with increased internet use among older adults (38). Moreover, older men often use the internet for information and communication purposes, while older women utilize it for social support (35). The number of children may also be a factor, with individuals having more children potentially having greater social support and relying less on online communication (35). Therefore, it is imperative to consider these variables in order to establish a more precise relationship between internet use and health.

In an effort to address potential biases from confounding variables, this research employs propensity score matching (PSM) to balance the characteristics of individuals who use the internet and those who do not. PSM is a statistical technique that matches individuals based on their probability of receiving a specific treatment or intervention, while accounting for the factors that affect this probability. By utilizing PSM, this study aims to reduce sample selection bias and improve the precision of determining the causal link between internet use and health results.

Furthermore, the impact of internet use on the health of empty-nest older adults in rural China and the underlying mechanisms are not yet fully understood. Social participation is widely acknowledged as a crucial factor that can significantly influence the health of older adults (39). Social participation involves engaging in activities that facilitate interaction with others, such as volunteering, participating in community events, joining social clubs, and attending religious services (40). These activities not only provide opportunities for social interaction and integration for older adults but also foster a sense of purpose and belonging, which can lead to improved health outcomes (41, 42).

The Social Capital Theory provides a theoretical framework for understanding how social participation can mediate the relationship between internet use and health outcomes. According to this theory, social capital refers to the resources embedded in social networks and relationships, which can be accessed and utilized to achieve certain goals (43). Internet use facilitates the accumulation and mobilization of social capital by expanding social networks, fostering trust, and promoting reciprocity. This enhanced social capital can then translate into improved health outcomes through various mechanisms (44). Studies have shown that social participation in older adults is associated with positive health benefits, including a reduced risk of depression (45, 46), enhanced cognitive function (47, 48), better physical health (49, 50), and increased longevity (51, 52). For example, Glass et al. (53) found that participating in social activities lowered the risk of dementia development in older adults. Similarly, Li et al. noted a connection between social participation and enhanced physical functioning as well as a decrease in chronic health conditions among the older adults (54).

The potential of internet use to promote social participation among older adults has been recognized (55). The internet serves as a platform for older individuals to connect with others, access information, and engage in social activities, irrespective of their physical location or mobility. A growing body of research suggests that internet use can not only enhance social participation but also improve health outcomes in older adults (56–58). For example, Cotten et al. (59) found that internet use was linked to increased social participation and decreased loneliness in older adults. Similarly, Chopik (90) discovered a positive association between internet use and social support and wellbeing in older adults (59). Furthermore, Shapira et al. found that internet use was correlated with improved cognitive function in older adults, potentially due to increased social participation and engagement (60). Therefore, it is hypothesized that internet use may promote health by enhancing social participation among empty-nest older adults in rural China, as facilitated by the accumulation and mobilization of social capital.

To address these challenges, this study aims to investigate the relationship between internet use and health outcomes in empty-nest older adults in rural China while controlling for confounding variables such as age, gender, education level, marital status, number of children, chronic diseases, smoking, drinking alcohol, physical activity, and children's support (35). Additionally, the study aims to explore the mediating role of social participation in this relationship. Using data from the China Health and Retirement Longitudinal Survey (CHARLS) 2020 and employing propensity score matching (PSM) to reduce sample selection bias, the research seeks to provide valuable insights into the potential health benefits of internet use for this population.

## 2 Method

### 2.1 Participants

The data for this study were collected from the China Health and Retirement Longitudinal Survey (CHARLS) conducted by the National Development Institute of Peking University (http://charls.ccer.edu.cn/charls/). CHARLS is a nationally representative longitudinal survey of individuals in China aged 45 and above, covering various aspects such as demographic background, family, health status, healthcare, work, retirement, income, expenditure, assets, and interviewer observations. The baseline national wave of CHARLS took place in 2011 and continued every 2 years. Data will be made public 1 year after collection (61). The study protocol was approved by the Ethical Review Committee of Peking University (IRB00001052-11015). Respondents were given a statement explaining the purpose of the study, and all study participants signed a written informed consent prior to being investigated. All methods will be carried out in accordance with relevant guidelines and regulations of the Declaration of Helsinki.

**Figure 1:**
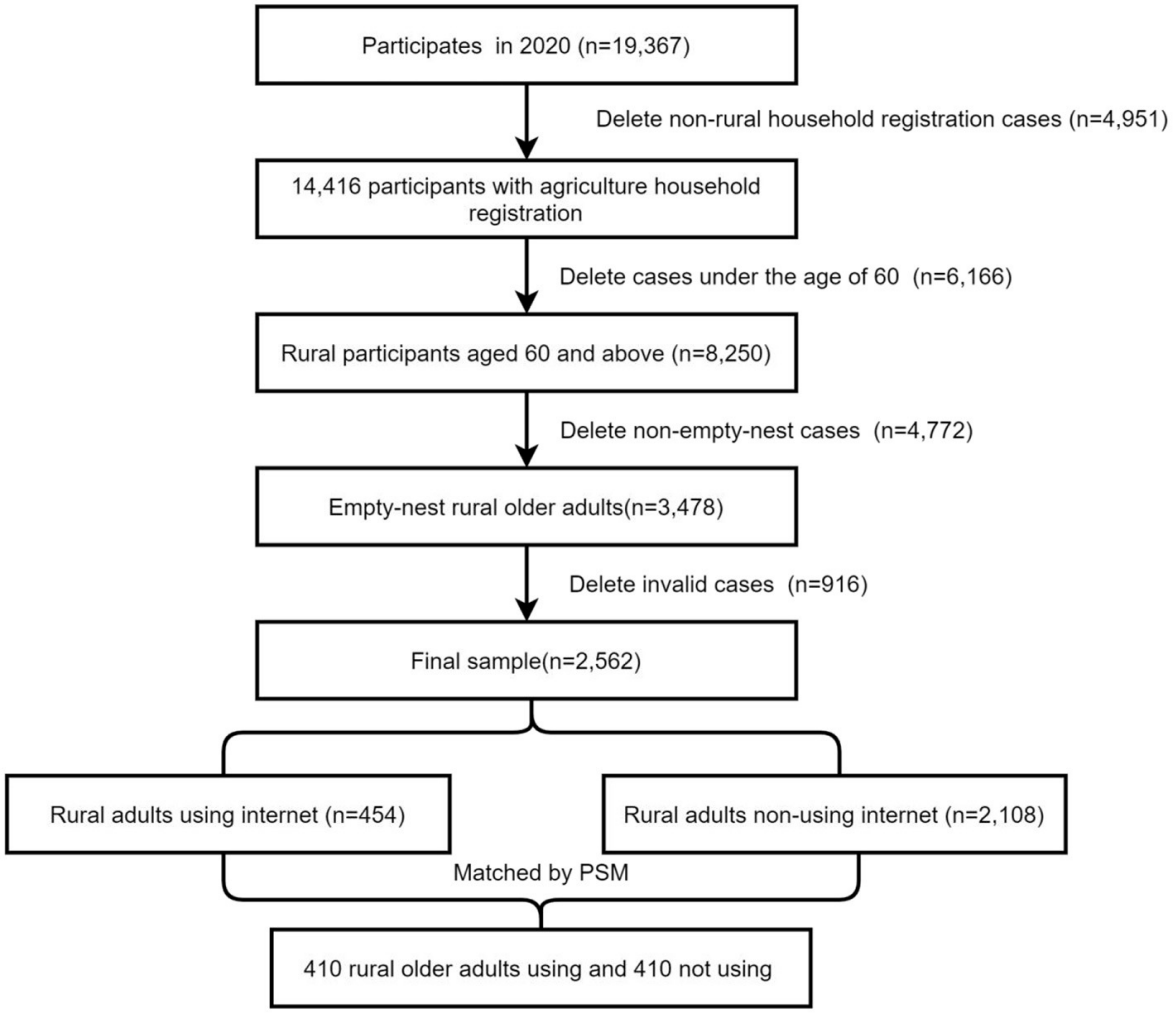
The construction of data. Source: Zhao et al. (91).

For our analysis, participants were selected from the latest 2020 data of CHARLS. In this study, empty-nest rural older adults were defined as individuals aged 60 and above who live in rural areas and do not have children or other family members living with them. Initially, 4,951 participants without rural household registration and 6,166 adults under 60 were excluded, leaving 8,250 rural participants aged 60 and above. Subsequently, 4,772 non-empty nest participants and 916 invalid cases (e.g., missing values on internet use) were removed, resulting in 2,562 subjects. Using PSM, 454 older adults in the internet use group were matched with 2020 older adults in the non-internet use group based on twelve covariates, resulting in 410 participants in each group (refer to Figure 1 for samples election).

### 2.2 Measures

#### 2.2.1 Independent variable

In this paper, Internet use is selected as the independent variable. According to the previous study, internet use was determined based on a question from the CHARLS 2020 Questionnaire, which asked respondents whether they had used the internet in the preceding month (61). Activities considered as being online included chatting on mobile networks, reading news, watching videos, playing games, and others. Respondents who answered “yes” were categorized as internet users, while those who answered “no” were categorized as non-internet users. This categorization was used to differentiate between older adults in rural areas who use the internet and those who do not.

#### 2.2.2 Outcome variable

##### 2.2.2.1 Emotional health

Depression, a prevalent emotional health concern among older adults, was evaluated in this research using a simplified version of the Center for Epidemiologic Studies Depression Scale (CES-D). The CES-D, a commonly used depression assessment tool in household surveys (62), has been validated for use in the Chinese population (63). In the CHARLS study, depression levels were measured through 10 items, each representing a different scenario related to depressive symptoms. Participants were asked to report how often they experienced these scenarios in the past week, with response options ranging from “almost none” (less than 1 day) to “most of the time” (5–7 days). Responses were scored from 0 to 3, with higher scores indicating higher levels of depression. The CES-D has good psychometric properties, with high internal consistency (Cronbach's alpha coefficients typically ranging from 0.85 to 0.90), good test-retest reliability (correlations typically ranging from 0.50 to 0.70), and strong construct validity (63–65). In our study, the Cronbach's alpha value for the CES-D 10 was 0.85, indicating good internal consistency and reliability of the measurement.

##### 2.2.2.2 Physical health

The physical health of participants in CHARLS was evaluated using the Activities of Daily Living (ADL) scale, a reliable and valid tool widely utilized in both China and internationally (66). This scale comprises 12 items that assess an individual's capability to carry out fundamental self-care activities such as dressing, bathing, eating, and managing money, among others. Participants rated their difficulty level for each item on a 4-point scale, ranging from “No difficulty” to “Cannot do it”, with higher scores indicating increased task difficulty. A cumulative score was calculated by summing the scores for all items, with a higher total score reflecting greater independence in ADL. Individuals reporting difficulty in any of the 12 items were categorized as having an ADL disability. Previous research has demonstrated that the Chinese version of the ADLscale exhibits strong internal consistency (Coefficient Alpha = 0.86) and good test-retest reliability (r = 0.90) (67). The ADLs scale in CHARLS has proven to be a valid measure of older adults' daily performance (68). Measurement of ADL functions is essential as it serves as an early sign of functional decline in old age (69). These measurements are also predictors of the need for alternative living arrangements, the utilization of paid home care, and admission to nursing homes (69). In the current sample, the ADLscale show good internal consistency (Coefficient Alpha = 0.83).

##### 2.2.2.3 Cognitive health

The evaluation of cognitive health in the CHARLS questionnaire uses the episodic memory and mental intactness sections from the Mini-Mental State Examination (MMSE) designed and developed by Folstein et al. (70). Episodic memory was assessed through immediate and delayed word recall tests, while mental intactness was measured using tasks such as numerical ability (serial subtraction of 7 from 100, five times), time orientation (including today's date, day of the week, month, year), and picture drawing (intersecting pentagon copying test). The mental intactness tasks were graded on a scale of 0 to 11, with higher scores indicating better cognitive function (71). Episodic memory performance was determined by participants' ability to recall a list of 10 words immediately and after a delay, with scores ranging from 0 to 10 for both instances (72). A composite cognition score was computed by adding the scores from the episodic memory and mental intactness tasks, resulting in a total score ranging from 0 to 31. Previous research has shown that the MMSE have strong internal consistency (Coefficient Alpha = 0.85–0.96) and good test-retest reliability (r = 0.80-0.85) (70, 73). These tests have been widely used and validated for measuring cognitive ability utilized in CHARLS (74–76).

##### 2.2.2.4 Social participation

Based on the previous studies, the social participation of respondents was assessed using the CHARLS questionnaire, which inquired about engagement in various activities over the past month. These activities included interacting with friends or neighbors, playing games like mahjong or cards, caring for sick or disabled adults, participating in physical activities like dancing or qigong, involvement in community organizations, volunteering, attending educational courses, and engaging in other social activities. A score of 0 was assigned to respondents who did not partake in any of these activities, while those who participated received a score corresponding to the number of activities they engaged in.

#### 2.2.3 Covariates

In this study, based on data from the 2020 CHARLS survey, eleven potential confounding covariates were identified: age, gender, education status, marital status, number of children, chronic disease, smoking, drinking alcohol, physical activity, children's emotional support, and children's financial support. Education level categories ranged from 1 (illiteracy) to 11 (doctoral degree/Ph.D.), while marital status was categorized as 0 (single) or 1 (partnered/married). Physical activity was assessed by the frequency of engaging in physical activity for at least 10 min per week. Children's emotional support was evaluated by the frequency of communication through various means when not residing with the participant. Children's financial support was measured by the amount of financial assistance received from children in the previous year.

### 2.3 Statistical analysis

In this study, PSM is a crucial statistical method used to mitigate sample-selection bias and mixed bias by pairing individuals in the treatment group with similar counterparts in the control group (77, 78). PSM analysis typically involves two main steps (77). The initial step entails computing the propensity score and then matching subjects based on this score. The propensity score, derived from a logit regression model, is utilized to match subjects from the Internet use group and non-usage group under comparable circumstances. It is estimated as follows:


$$\begin{array}{l} P=P\left(D=1|x\right)=\frac{1}{1+e^{-\beta_{0}-\sum \beta_{i}x_{i}}} \end{array}$$

In the above formula, P represents the probability of subjects using internet, D = 1 represents subjects in the using internet group, and X refers to covariates.

The secondly analysis is to estimate the Average Treatment effect on the Treated (ATT) by the following model, which reflects the effect of using internet on the emotional, physical and cognitive health of older adults. The significance of ATT is tested by a paired *t*-test (79).


$$\begin{array}{r} ATT=E\left(Y_{1}|D=1\right)-E\left(Y_{0}|D=1\right)=E\left(Y_{1}|D=1,P\right)\\ -E\left(Y_{0}|D=0,P\right) \end{array}$$

In the above formula, Y_1_ and Y_0_ are the dependent variables of the matched samples in the using internet and non-using internet groups respectively. P is the propensity value, D=0 indicates the subjects non-using internet group, and D=1 indicates subjects with using internet. As Y_0_|D = 1 cannot be directly observed, the establishment of the above model must satisfy the “unconfoundedness assumption”, which requires to control factors associating with older adults' using internet group. In order to test the robustness of PSM, various matching methods are generally used. In our study, we used four matching methods: nearest neighbor matching, kernel matching, optimal matching and radius matching in all.

Finally, to examine the mediating role of social participation in the relationship between Internet use and health outcomes, a mediation analysis might have been conducted. This analysis would involve assessing the direct and indirect effects of Internet use on emotional and physical health, with social participation as the mediator.

To compare differences between the using internet group and the non-using internet group, the independent sample *t*-test and Chi-square test were employed. The significance level for all tests was set at 0.05. PSMATCH2 and NNMATCH in Stata 14.0 were performed to conduct the statistical analysis.

## 3 Results

### 3.1 Descriptive statistics of each variable

The study includes a total of 16 variables, comprising three outcome variables, one independent variable, and 12 covariates. Table 1 presents the item coding, measurement units, and descriptive statistics for each variable.

**Table 1:** Descriptive statistics of each variable.

**Variables**	**Minimum**	**Maximum**	**Mean**	**Standard deviation**	**Item code/unit of measurement**
**Dependent variable**					
Physical health	14	48	46.212	3.815	-
Cognitive health	3	30	17.676	4.298	-
Emotion health	0	30	9.766	6.682	-
**Independent variable**					
Internet use	0	1	0.180	0.382	0-no,1-yes
**Covariates**					
Age	60	92	69.193	6.018	Years
Gender	0	1	0.500	0.500	0-female,1-male
Education level	1	9	2.830	1	1-illiterate,9-Bachelor's Degree
Marital status	0	1	0.840	0.369	0-single,1-partnered/married
Number of children	0	9	2.754	1.315	-
Chronic disease	0	1	0.368	0.482	0-no,1-yes
Smoking	0	1	0.270	0.445	0-no,1-yes
Drinking alcohol	0	1	0.340	0.475	0-no,1-yes
Physical activity	0	7	2.880	1.878	Days per week
Children's emotional support	0	9	5.625	2.903	Contact frequency
Children's financial support	0	12	5.524	3.881	Logarithm of amount
Social participation	0	5	0.344	0.625	Number of social activities

### 3.2 Covariates' balancing test before matching

Covariate balancing tests were conducted between groups of older adults based on internet use. Table 2 displays significant differences in 11 covariates, with the exception of chronic disease, between the two groups. The internet user group exhibited higher levels of education, social participation, physical activity, and other factors compared to the non-internet user group. These notable variations should be taken into account when assessing the relationships between internet use and the emotional, physical, and cognitive health of older adults.

**Table 2:** Covariates' differences test between using internet group and not using internet group before matching.

**Variables**	**Using internet group (*****N*** = **454)**		**Not using internet group (*****N*** = **2,108)**		***t/***χ***^**2**^***		
	**Mean**	**SD**	**Mean**	**SD**	Δ**Mean**	***t***	***p***
Age	65.597	4.285	69.968	6.056	−4.371	−14.60	< 0.001
Education level	3.852	1.717	2.608	1.602	1.245	14.82	< 0.001
Social participation	0.590	0.824	0.290	0.560	0.300	9.428	< 0.001
Physical activity	3.286	1.775	2.793	1.889	0.492	5.092	< 0.001
Numbers of children	2.544	1.174	2.799	1.340	−0.255	−3.754	< 0.001
Children's financial support	5.865	3.897	5.45	3.875	0.415	2.070	0.039
Children's emotional support	6.242	2.791	5.491	2.91	0.751	5.022	< 0.001
	**Percent**		**Percent**		Δ**percent**	χ*^2^*	***p***
	**0**	**1**	**0**	**1**	**1**		
Gender	187 (41.2)	267 (58.8)	1,092 (51.8)	1,016 (48.2)	10.6	16.831	< 0.001
Marital status	42 (9.3)	412 (90.7)	373 (17.7)	1,735 (82.3)	8.4	19.618	< 0.001
Chronic disease	285 (62.8)	169 (37.2)	1,334 (63.3)	774 (36.7)	0.5	0.041	0.839
Smoking	306 (67.4)	148 (32.6)	1,561 (74.1)	547 (25.9)	6.7	8.357	0.004
Drinking alcohol	263 (57.9)	191 (42.1)	1,421 (67.4)	687 (32.6)	9.5	14.905	< 0.001

Gender: 0-female, 1-male; Marital status: 0-single, 1-partnered/married; Chronic disease: 0-having no chronic disease, 1-having at least one chronic disease; Smoking: 0-no smoking, 1-not smoking; Drinking alcohol: 0-not drinking alcohol, 1-drinking alcohol.

### 3.3 Propensity score matching analysis

A logistic regression model was conducted with internet use as the output variable and twelve covariates as predictor variables. Specifically, age (*p* < 0.01), education level (*p* < 0.01), physical activity (*p* = 0.017), children's emotional support (*p* = 0.02), children's financial support (*p* = 0.010), and social participation (*p* < 0.01) were found to be significant predictors of older adults' internet use (as shown in Table 3). Subsequently, these regression results were utilized to develop a prediction model that calculates the likelihood of an older adult using the internet. A higher propensity score indicates a greater likelihood of internet use among older adults.

**Table 3:** Logistic regression estimates of using internet among rural empty-nest older adults.

**Variables**	**Coefficient**	**SE**	**Z**	***P***	**[95% CI]**
Age	−0.150	0.014	−10.760	< 0.001	[−0.177, −0.122]
Gender	0.007	0.153	0.050	0.963	[-0.292, 0.306]
Education status	0.354	0.037	9.670	< 0.001	[0.282, 0.425]
Marital status	0.064	0.192	0.330	0.741	[-0.314, 0.441]
Number of children	0.032	0.057	0.570	0.570	[-0.079, 0.144]
Chronic disease	−0.014	0.119	−0.120	0.908	[-0.248, 0.220]
Smoking	−0.013	0.145	−0.090	0.931	[-0.296, 0.271]
Drinking alcohol	0.175	0.131	1.340	0.181	[-0.081, 0.432]
Physical activity	0.073	0.031	2.380	0.017	[0.013, 0.133]
Children's emotional support	0.070	0.023	3.070	0.002	[0.025, 0.114]
Children's financial support	0.041	0.016	2.580	0.010	[0.010, 0.072]
Social participation	0.463	0.081	5.700	< 0.001	[0.304, 0.623]
Constant	6.166	1.032	5.970	< 0.001	[4.143, 8.188]
Pseudo R^2^	0.188				
LR chi^2^ (df)	449.07^***^ (12)				

CI, Confidence Interval. ^***^p < 0.001.

### 3.4 Matching and balancing test

**Figure 2:**
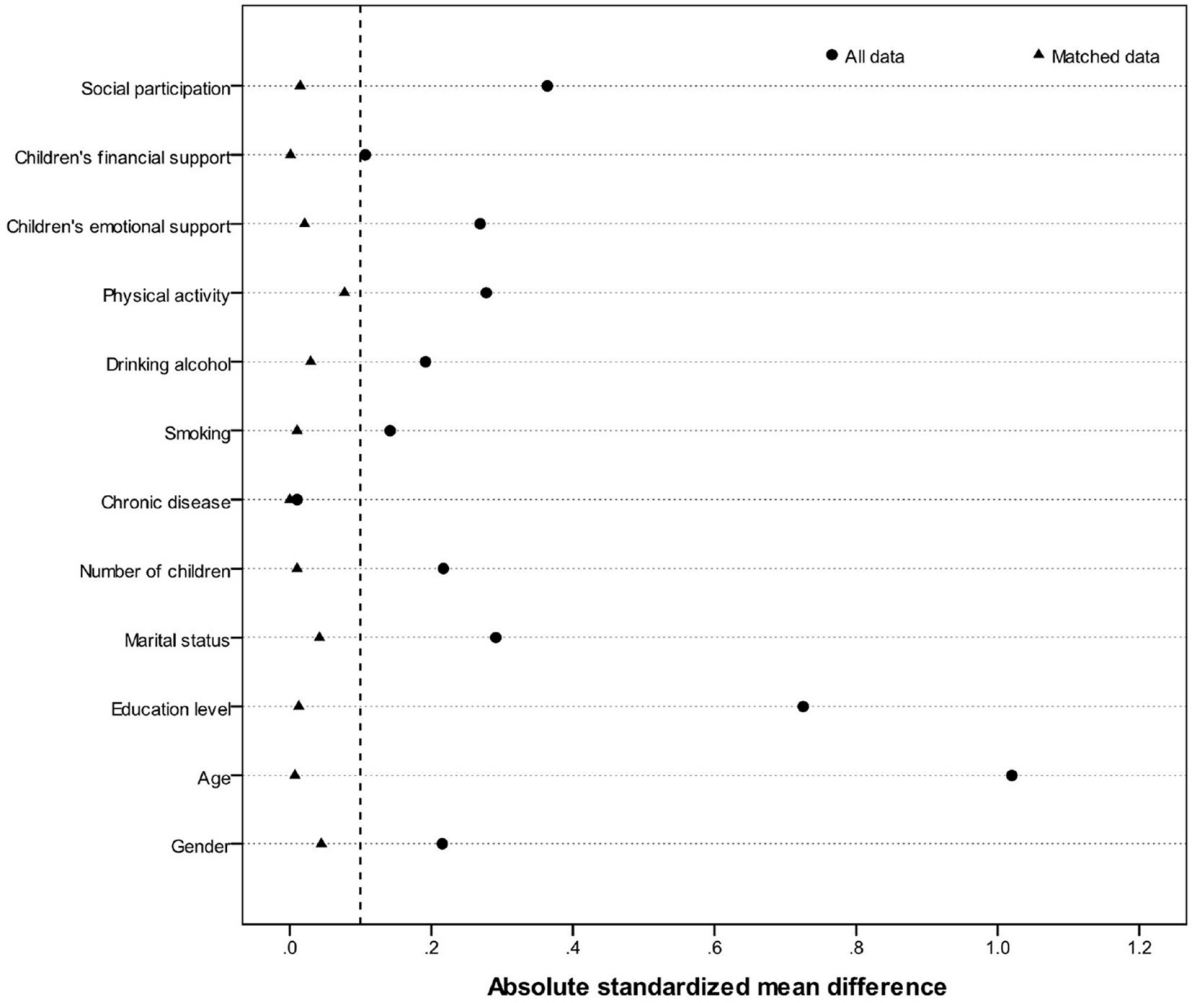
The change in the absolute value of the standard deviation of the covariates before and after the matching of 12 covariates.

The propensity score was calculated to match 454 older adults who use the internet with 2,018 older adults who do not use the internet. The nearest-neighbor matching method (1:1) was employed, resulting in a total of 410 pairs of successfully matched samples. Following matching, the independent sample *t*-test for each covariate between the internet-using and non-using groups no longer showed significant differences (*p* > 0.05) (as shown in Table 4). Figure 2 displays the absolute values of standardized mean differences before matching (circles) and after matching (triangles). With the exception of the chronic disease covariate, the standard deviations of all covariates substantially improved after matching, with absolute values below 0.1, indicating that the assumption of covariate balance was met.

**Table 4:** Covariates' balancing test between using internet group and not using internet group after matching.

**Variables**	**Using internet group (*****N*** = **410)**		**Not using internet group (*****N*** = **410)**		***t/***χ***^**2**^***		
	**Mean**	**SD**	**Mean**	**SD**	Δ**Mean**	***t***	***p***
Age	65.949	4.313	65.98	4.266	−0.032	−0.106	0.916
Education level	3.685	1.687	3.707	1.715	−0.022	−0.185	0.853
Social participation	0.515	0.731	0.502	0.721	0.012	0.241	0.810
Physical activity	3.208	1.745	3.071	1.869	0.137	1.088	0.277
Numbers of children	2.527	1.179	2.515	1.119	0.012	0.152	0.879
Financial support	5.879	3.904	5.884	3.811	−0.004	−0.016	0.987
Emotional support	6.090	2.855	6.149	2.62	−0.059	−0.306	0.760
	**Percent**		**Percent**		Δ**Percent**	*χ^2^*	***p***
	**0**	**1**	**0**	**1**	**1**		
Gender	175 (42.7)	235 (57.3)	166 (40.5)	244 (59.5)	−2.2	0.407	0.524
Marital status	41 (10.0)	369 (90.0)	46 (11.2)	364 (88.8)	1.2	0.321	0.571
Chronic disease	255 (62.2)	155 (37.8)	255 (62.2)	155 (37.8)	0	0.017	0.924
Smoking	276 (67.3)	134 (32.7)	274 (66.8)	136 (33.2)	−0.5	0.022	0.882
Drinking alcohol	245 (59.8)	165 (40.2)	239 (58.3)	171 (41.7)	−1.5	0.182	0.670

### 3.5 Common support assumption test

**Figure 3:**
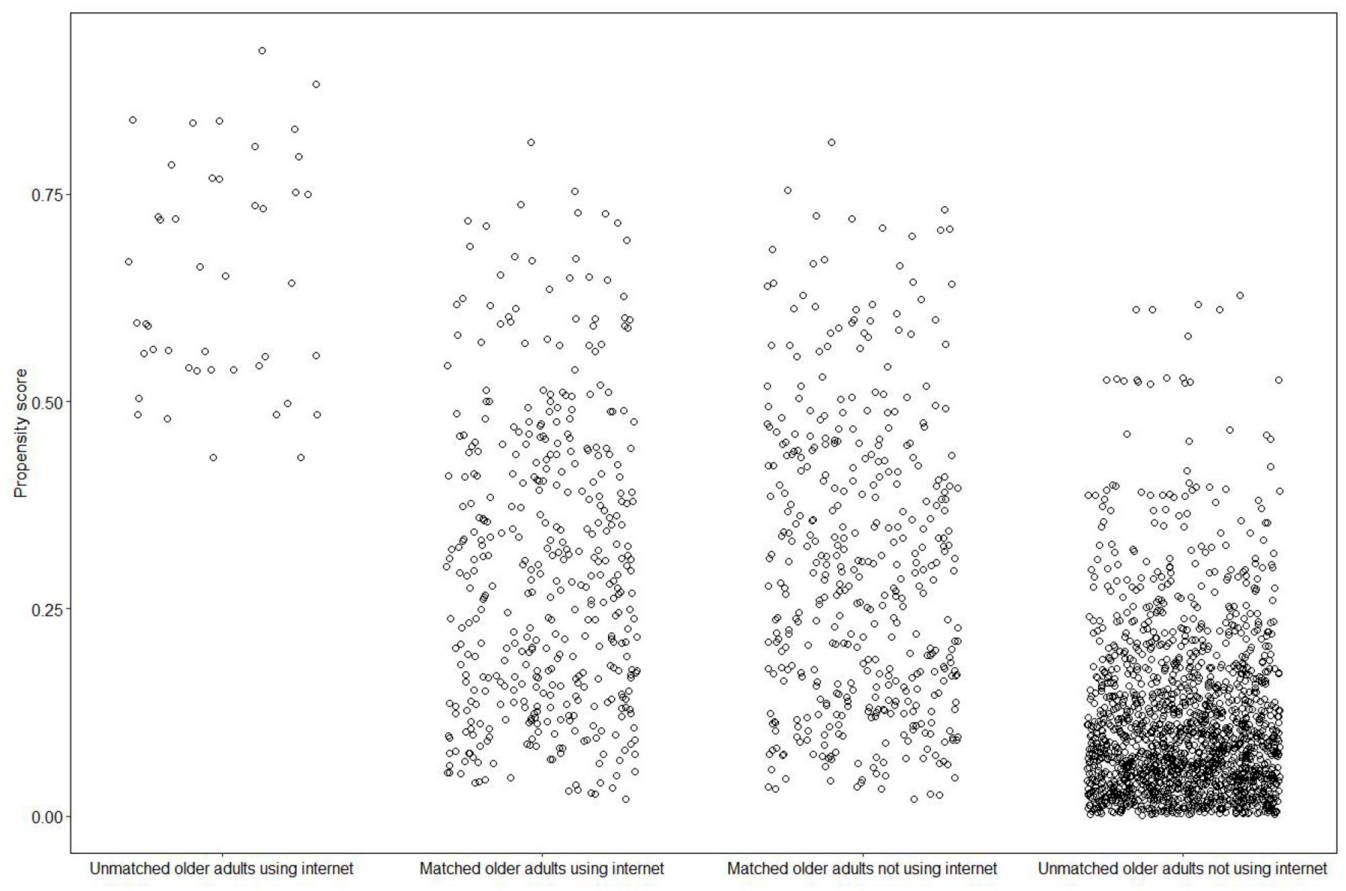
Distribution of the propensity scores.

The common support hypothesis, as proposed by Rosenbaum and Rubin, posits that the propensity score ranges of groups using the internet and not using the internet should be similar (78). In Figure 3, the distribution of propensity scores for four groups is illustrated. Following the matching process, 44 older adults using the internet and 1,698 older adults not using the internet were excluded, as depicted in the first and fourth columns of Figure 3, respectively. The scatterplots of propensity scores for matched older adults using and not using the internet were nearly identical, as shown in the second column compared to the third column in Figure 3, indicating that the common support assumption was met.

### 3.6 Average treatment effect on the treated

The bootstrap method was utilized in this study to estimate the average treatment effect on the treated (ATT) and empirical standard error post propensity score matching. The findings indicate that older adults who use the internet exhibited significantly lower levels of depression (*p* < 0.01) and higher physical health (*p* < 0.05) compared to older adults who do not use the internet, after controlling for confounding factors through PSM. However, there was no significant difference in cognitive health between the two groups. Specifically, depression levels decreased significantly by 0.795 and physical health improved significantly by 0.433 (refer to Table 5). Overall, internet use was associated with a 0.928% improvement in physical health and an 8.659% reduction in depression levels compared to non-users.

**Table 5:** ATT of using internet on rural empty-nest older adults' health.

**Dependent variable**	**Using internet**	**Not using internet**	**ATT**	**SE**	***t***	***p***
ADL	47.071	46.638	0.433	0.169	2.562^**^	0.010
Depression	8.386	9.181	−0.795	0.289	−2.751^**^	0.005
Cognitive health	17.924	17.688	0.236	0.193	1.218	0.223

(1) SE-standard error; ATT-average treatment effect on the treated; (2) ^**^p < 0.01.

### 3.7 Robustness test

To verify the robustness of the nearest neighbor matching method (1:1), we used optimal matching, kernel matching using normal density, and radius (caliper = 0.05) to conduct the PSM. The results of three matching methods were consistent (see Table 6), indicating that the estimation results of 1:1 non repetitive nearest neighbor matching were robust.

**Table 6:** The results of sensitivity analysis.

**Gamma**	**Optimal**			**Kernel**			**Radius**		
	**ATT**	**SE**	**t**	**ATT**	**SE**	**t**	**ATT**	**SE**	**t**
ADL	0.449	0.163	2.755^**^	0.515	0.171	3.012^**^	−0.518	0.181	−2.862^**^
Depression	−0.743	0.289	−2.571^*^	−0.865	0.344	−2.515^*^	−0.863	0.381	−2.265^*^
Cognitive health	0.099	0.175	0.566	0.163	0.222	0.734	0.172	0.228	0.754

^*^p < 0.05, ^**^p < 0.01.

### 3.8 Social participation's mediating effect analysis

The results of propensity score matching analysis revealed a positive impact of internet use on both physical and emotional health. To investigate the potential mediating role of social participation in these relationships, separate regression analyses were conducted for physical health (model 1), emotional health (model 2), and social participation (model 3), with other variables serving as covariates. The findings showed that all three regression models were statistically significant. internet use was found to significantly predict emotional health and social participation, but not physical health. On the other hand, social participation was a significant predictor of physical health but not emotional health. Detailed results can be found in Table 7.

**Table 7:** Regression analysis among variables.

**Model**	**Outcome variable**	**Predictor variable**	**Model fit**		**Significance**	
			***R** ^2^*	***F***	***_**β*	***t***
Model 1	Physical health	Internet	0.080	16.972^***^	0.396	1.913
		Social participation			0.284	2.382^*^
		11 covariates			-	-
Model 2	Depression	Internet	0.076	16.128^***^	−0.910	−2.504^*^
		Social participation			−0.300	−1.431
		11 covariates			-	-
Model 3	Social participation	Internet	0.057	12.710^***^	0.223	6.554^***^
		11 covariates			-	-

^*^p < 0.05, ^***^p < 0.001.

To compare the direct and indirect effects of internet use on physical and emotional health, we conducted an analysis using 5,000 bootstrap samples. The indirect effect of internet use on physical health through social participation was found to be significant at a 95% confidence interval (Indirect effect = 0.064, L.L. = 0.024, U.L. = 0.111), while the direct effect of internet use was deemed insignificant (Indirect effect = 0.396, L.L. = −0.010, U.L. = 0.802). Similarly, the indirect effect of internet use on emotional health through social participation was found to be insignificant at a 95% confidence interval (Indirect effect = −0.067, L.L. = −0.168, U.L. = 0.138), whereas the direct effect of internetuse was significant (indirect effect = −0.910, L.L. = −1.623, U.L. = −0.197). Detailed results can be found in Table 8.

**Table 8:** The direct and indirect effects of using internet on physical and emotional health.

**Effect**	**Physical health**				**Emotional health**			
	**Size**	**S.E**.	**95% CI**		**Size**	**S.E**.	**95% CI**	
			**L.L**.	**U.L**.			**L.L**.	**U.L**.
Direct effect	0.396	0.207	−0.010	0.802	−0.910	0.364	−1.623	−0.197
Indirect effect	0.064	0.022	0.024	0.111	−0.067	0.044	−0.168	0.138

L.L., lower limit; U.L., upper limit.

## 4 Discussion

The study delved into the impact of internet use on the emotional, physical, and cognitive health of empty-nest older adults in rural China, with a focus on the mediating role of social participation. Findings indicated that internet use was linked to enhanced emotional and physical health, though not cognitive health. Moreover, social participation was found to partially mediate the relationship between internet use and physical health, but not emotional health.

Several specific findings emerged. Firstly, the findings of this study suggest that empty-nest older adultsusing the internet showed lower levels of depression compared to those not using the internet, after adjusting for confounding factors. This result is consistent with the theory of social interaction, which posits that engaging in social interactions can strengthen relationships and ultimately help reduce depressive symptoms in older adults (80). It is also in line with previous research that has found a positive association between internet use and emotional wellbeing in older adults (29, 81). For example, a study conducted in America found that internet use was associated with a decrease in depressive symptoms among older adults (30).

Secondly, consistent with prior research, this study also highlights the positive impact of Internet use on the physical health of empty-nest older adults in rural China (82). By providing access to health-related information and resources, the internet enables these individuals to enhance their knowledge on disease prevention, nutrition, and exercise, ultimately leading to improvements in their physical wellbeing (83, 84). Additionally, the internet fosters social connections, mitigating feelings of loneliness and isolation commonly experienced by this demographic, thereby benefitting both their mental and physical health (9, 12). It is worth noting that the influence of internet use on older adults' health outcomes may vary based on factors such as digital literacy, the quality of online health information accessed, and socioeconomic status. Hence, while the study corroborates existing literature, it is essential to take these factors into account when interpreting the results.

Thirdly, the study results indicated no significant correlation between internet use and cognitive health in rural Chinese older adults who are empty-nesters. This finding contradicts previous research suggesting a positive link between internet use and cognitive health in older adults (85). For instance, a study in China showed that internet use was linked to enhanced cognitive function in the older adults (86). The discrepancies in these findings may stem from insufficient control over confounding variables in earlier studies. In this study, propensity score matching was used to address sample selection bias and control for confounding factors, possibly explaining the lack of significant results. This underscores the importance of rigorous methodological approaches when examining the relationship between internet use and cognitive health in older adults.

Fourthly, the findings of this study indicate that social participation partially mediates the relationship between internet use and physical health, but not emotional health, among empty-nest older adults in rural China. This aligns with previous research showing a positive link between internet use and social engagement in older populations (56). For instance, a study conducted in the United States demonstrated that internet use was connected to increased social participation among older adults (87). Moreover, past studies have highlighted the positive correlation between social participation and physical health in older individuals (39). For example, a study in China revealed that social engagement was linked to better self-rated health in older adults (88). However, the benefits of internet use on emotional wellbeing can be direct and independent of social participation (89). This suggests that the internet can positively impact emotional health through avenues such as access to information, entertainment, online support groups, and educational resources. These benefits can foster a sense of purpose, self-efficacy, and positive mood, all of which contribute to emotional wellbeing. While social participation remains crucial for emotional health, the positive effects of internet use on emotional wellbeing are not solely dependent on social interactions, indicating the presence of other beneficial mechanisms. Therefore, promoting internet use could be a valuable strategy for enhancing emotional wellbeing, even if social participation is not the primary focus.

Finally, this study has several limitations that should be acknowledged. Firstly, although our study found that internet use can improve the emotional and physical health of rural empty-nest older adults, with social participation acting as a mediator in the relationship between internet use and physical health, the causal validity of this relationship remains to be verified. Our study is cross-sectional in nature, which limits our ability to establish causal relationships between internet use, social participation, and health outcomes. Longitudinal studies are needed to track changes in internet use, social participation, and health over time and to better understand the directionality of these relationships. Secondly, the measures of emotional, physical, and cognitive health used in this study are self-reported, which may be subject to bias. Thirdly, the digital divide remains a significant challenge, particularly in rural areas, and may limit the potential benefits of internet use. While we employed PSM to balance the characteristics of internet users and non-users, the digital divide may still influence the generalizability of our findings. Future research should explore ways to bridge this divide and ensure that the benefits of internet use are accessible to all older adults, regardless of their socio-economic status or geographical location. Furthermore, while we considered a range of variables as covariates, several important factors, such as socio-economic status (SES), were not included in our analysis. Therefore, future research should explicitly incorporate SES and other relevant variables as key covariates to enhance our understanding of their role in the relationship between internet use, social participation, and health outcomes among rural empty-nest older adults. Lastly, our study did not differentiate between types of internet use or consider the frequency and duration of usage, both of which may impact health outcomes differently. Future research should examine these factors more closely to understand how specific online behaviors affect health.

## 5 Implications

The study's findings carry significant implications for enhancing the wellbeing of empty-nest older adults in rural China. The results indicate that utilizing the internet could positively impact the emotional and physical health of this demographic, with social engagement potentially acting as a mediator in this association. As such, interventions targeting increased internet use and social participation among empty-nest older adults in rural China could prove beneficial for their overall health. It is worth noting, however, that the study's cross-sectional design precludes definitive causal conclusions. Future research employing longitudinal approaches is necessary to establish the causal link between internet use, social participation, and health outcomes in this population.

## 6 Conclusion

In conclusion, this study suggests that internet use can contribute to improved emotional and physical health among empty-nest older adults in rural China, with social participation playing a mediating role in the relationship between internet use and physical health. Further research is needed to explore the potential mechanisms underlying these associations and to develop targeted interventions aimed at promoting internet use and social participation among this population.

## Data Availability

The original contributions presented in the study are included in the article/supplementary material, further inquiries can be directed to the corresponding authors.
